# Extracellular Vesicles as a Means of Viral Immune Evasion, CNS Invasion, and Glia-Induced Neurodegeneration

**DOI:** 10.3389/fncel.2021.695899

**Published:** 2021-07-05

**Authors:** Miranda D. Horn, Andrew G. MacLean

**Affiliations:** ^1^Neuroscience Program, Brain Institute, Tulane University, New Orleans, LA, United States; ^2^Division of Comparative Pathology, Tulane National Primate Research Center, Tulane University, Covington, LA, United States; ^3^Department of Microbiology & Immunology, Tulane University School of Medicine, New Orleans, LA, United States; ^4^Tulane Center for Aging, New Orleans, LA, United States

**Keywords:** extracellular vesicles, exosomes, microvesicles, virus, glia, neurodegeneration, microglia, astrocyte

## Abstract

Extracellular vesicles (EVs) are small, membrane-bound vesicles released by cells as a means of intercellular communication. EVs transfer proteins, nucleic acids, and other biologically relevant molecules from one cell to another. In the context of viral infections, EVs can also contain viruses, viral proteins, and viral nucleic acids. While there is some evidence that the inclusion of viral components within EVs may be part of the host defense, much of the research in this field supports a pro-viral role for EVs. Packaging of viruses within EVs has repeatedly been shown to protect viruses from antibody neutralization while also allowing for their integration into cells otherwise impervious to the virus. EVs also bidirectionally cross the blood-brain barrier (BBB), providing a potential route for peripheral viruses to enter the brain while exiting EVs may serve as valuable biomarkers of neurological disease burden. Within the brain, EVs can alter glial activity, increase neuroinflammation, and induce neurotoxicity. The purpose of this mini-review is to summarize research related to viral manipulation of EV-mediated intercellular communication and how such manipulation may lead to infection of the central nervous system, chronic neuroinflammation, and neurodegeneration.

## Introduction

Originally thought to be used strictly as a waste removal system (Johnstone et al., 1991), extracellular vesicles (EVs) have since been shown to be involved in complex intercellular communication, transferring proteins, nucleic acids, and other biologically relevant molecules between cells. Within the central nervous system (CNS), EVs can even have neuroprotective effects. For example, oligodendrocytes support axonal transport and maintenance *via* EV secretion (Frühbeis et al., 2020), while neuron-derived EVs increase Aß uptake by microglia which may play a role in preventing the formation of amyloid plaques (Yuyama et al., 2012). EVs come in a range of types with different sizes and functions, with exosomes, microvesicles, and apoptotic bodies being the most commonly studied (for review see Yáñez-Mó et al., 2015). For the purposes of this mini-review, we will use the all-encompassing term EV to represent all types.

EVs have also been implicated in disease. Within the cancer field, EVs are associated with immune modulation and tumor progression (for review see Marar et al., 2021). EVs are also associated with various neurodegenerative diseases, including Alzheimer’s, Parkinson’s, and prion diseases (Thompson et al., 2016). EVs have also been implicated in latent viral infections. EVs from HIV-1 infected cells were capable of reactivating HIV-1 in latently infected cells, even when those EVs came from cells infected with defective HIV-1 (Arenaccio et al., 2015). This suggests a mechanism by which latent HIV-1 reservoirs could be reactivated in individuals receiving combined antiretroviral therapy. In a recent review of the role of EVs in Ebola virus infection, several Ebola viral proteins were present within EVs, suggesting a possible mechanism for viral latency seen in a portion of those infected (Pleet et al., 2019). However, most studies related to the role of EVs in viral infection to date have been done in the periphery, leaving many questions about the of role EVs in viral infections of the CNS left unanswered.

## EVs as A Means of Viral Immune Evasion

Numerous studies have shown that viruses or viral proteins can be packaged into EVs, allowing evasion of the immune response. JC polyomavirus (JCPyV), which is responsible for causing progressive multifocal leukoencephalopathy in immunocompromised individuals, is packaged into EVs and, when associated with EVs, able to evade neutralization by anti-JCPyV antisera (Morris-Love et al., 2019; O’Hara et al., 2020). Similar viral association with EVs and evasion of neutralization has been shown in herpes simplex virus 1 (Bello-Morales et al., 2018), hepatitis E virus (Nagashima et al., 2014), hepatitis C virus (Ramakrishnaiah et al., 2013), and enterovirus 71 (Mao et al., 2016; Too et al., 2016). Hepatitis A virus (HAV) was shown to rely on VPS4B and ALIX, components of the exosomal ESCRT pathway, to mediate envelopment in a membranous structure that resembled an EV (Feng et al., 2013). This process allowed HAV to resist antibody neutralization and may explain how the virus remains active in hosts with anti-HAV antibodies present in their serum. However, the fact that enveloped HAV did not depend on the entire ESCRT pathway suggests that HAV acquires a membranous envelope in a mechanism distinct from that of EVs.

Many viruses, including SARS-CoV-2, enter cells using the host cell endosomal pathway (Shang et al., 2020). Once inside the cells, the viruses can hijack this pathway, and exosomes can then include multiple viral components. While beyond the scope of this particular mini-review, several viral proteins upregulate components of the ESCRT machinery and CD63 leading to potentially increased numbers of EVs being released from the target cells. These EVs may contain miRNAs and trans-activation response (TAR) RNA that can inhibit particular anti-inflammatory responses (Pleet et al., 2016) and simultaneously increase cellular activation (Barclay et al., 2017) in immune cells, respectively. This was exquisitely reviewed in detail recently by Jia et al. (2021).

Beyond direct immune evasion, research shows that EVs from virus-infected cells are also capable of modifying the immune response. While some studies have shown that EVs from virus-infected cells can enhance the host’s immune response against the virus (Walker et al., 2009; Cypryk et al., 2017), most research suggests that EVs from virus-infected cells manipulate the immune response in favor of increased viral infection. For example, treatment of mice with EVs from cells infected with hepatitis B virus reduced the number of infiltrating mononuclear cells, suggesting EVs from infected cells can suppress the innate immune response (Kakizaki et al., 2020). Additionally, nasopharyngeal carcinoma cells, which are positive for Epstein-Barr virus (EBV), were found to release EVs containing galectin-9 and latent membrane protein-1, both of which have been associated with inhibition of T-cell proliferation (Keryer-Bibens et al., 2006). This suggests a mechanism by which EVs from EBV+ nasopharyngeal carcinoma cells suppress the immune response and may support tumor growth. Similarly, various cell types infected with EBV have been shown to release EVs that suppress T-cell function (Dukers et al., 2000; Flanagan et al., 2003).

Another mechanism by which viruses may evade the immune response is through altering the content of EVs. In fact, several viral proteins have been found to do just that. For example, the HIV-1 protein Nef was shown to be released in EVs from HIV- and SIV-infected cells (McNamara et al., 2018). These Nef-containing EVs were able to transfer Nef to uninfected cells, providing a mechanism by which HIV/SIV infection may impact cells that are usually considered resistant to viral infection. Nef has also been shown to decrease the levels of CD4 in EVs secreted by HIV-infected CD4+ T cells (Carvalho et al., 2014). This decreased the ability of CD4+ T-cell EVs to inhibit HIV infection. In the context of EBV, the viral protein latent membrane protein-1 was shown to alter the protein and microRNA contents of EVs released from infected cells (Nkosi et al., 2020). Further, the altered EVs had an effect on several aspects of the target cells, including proliferation, adhesion, and migration. Together, the above evidence demonstrates various mechanisms by which viruses utilize the EV communication system to evade immune clearance and increase the viral spread.

## EVs as A Means for Expanded Viral Tropism

In addition to EVs being a means to evade and alter the immune response, they can also increase the infectivity of naïve cells. EVs containing RNA from enterovirus 71 are capable of establishing infection in cells at comparable levels to free virus (Mao et al., 2016). Similarly, Zhou et al. (2018) show that Langat virus is released from tick embryonic ISE6 cells *via* EVs that are capable of transmitting the virus to human cells. Further, as previously noted, the Nef-induced decrease in CD4 content of EVs was associated with an increased rate of HIV-1 infection of CD4+ T cells (Carvalho et al., 2014). Similarly, EVs from HIV-1 infected dendritic cells were shown to increase the transmission of HIV-1 to T cells as compared to the free virus (Kulkarni and Prasad, 2017). Further, rotovirus was found to be released in EVs in stool which was shown to be more infectious than free virus both *in vitro* and *in vivo*, increasing the multiplicity of infection in the gastrointestinal tract five-fold in comparison to free virus (Santiana et al., 2018).

Viral association with EVs has also been shown to expand viral tropism. When JCPyV is packaged into EVs it is capable of transferring the infection to cells that are otherwise impervious to infection due to a lack of viral receptors (Morris-Love et al., 2019; O’Hara et al., 2020). The packaging of herpes simplex virus-1 into EVs also allows for infection of cells that are otherwise unreceptive to herpes simplex virus-1 infection (Bello-Morales et al., 2018). Similarly, Bukong et al. (2014) found hepatitis C viral RNA within EVs released from hepatitis C virus-infected cells and found that these EVs could confer active infection to hepatocytes in a receptor-independent fashion. Contrastingly, norovirus was released in EVs that were infectious, but the infectivity of these EVs was dependent on the murine norovirus-1 receptor (Santiana et al., 2018). Beyond EVs serving as a means to increase cellular tropism of viruses, they may also serve to increase tissue tropism, leading to infection of the CNS.

## EVs as A Means of CNS Infection

**Figure 1:**
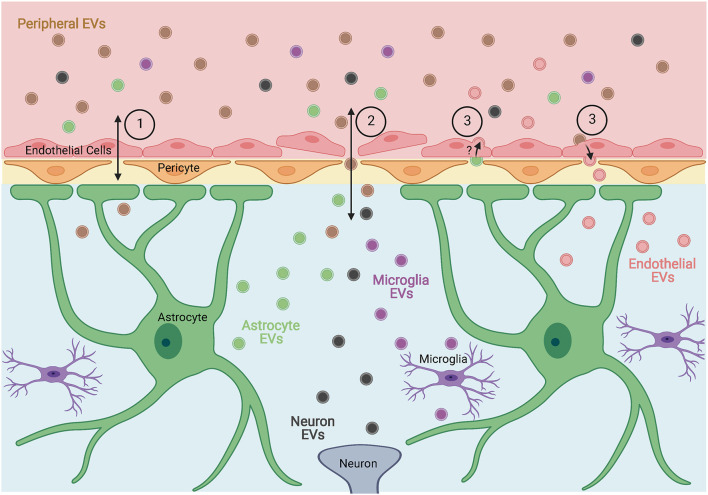
Proposed mechanisms by which extracellular vesicles (EVs) cross the blood-brain barrier (BBB). EVs, or their contents, can enter or exit the brain through (1) receptor-mediated or adsorptive transcytosis, (2) breakdown of the BBB allowing for direct passage between endothelial cells, and (3) entry into endothelial cells and subsequent alteration of endothelial EVs. Figure created with BioRender.com.

In a study of EVs derived from seven human and three murine cell lines, Banks et al. (2020) found that all the EVs tested were capable of crossing the blood-brain barrier (BBB) of mice. Additionally, the olfactory bulb, cortex, and cerebellum appeared to have relatively equal uptake of EVs in most cases, with the olfactory bulb showing significantly higher uptake for four of the EV types tested. The mechanisms by which EVs crossed the BBB appeared to vary across cell types, suggesting multiple methods for EV entry into the CNS exist (Figure 1). In a separate study, it was shown that EVs isolated from the serum of lipopolysaccharide-treated or high-fat diet mice were able to activate microglia and astrocytes when injected peripherally in healthy mice, suggesting that these EVs not only cross the BBB but also impact resident cells (Li et al., 2018). Similarly, EVs isolated from the serum of two different models of peripheral inflammation were able to cross the BBB and induce neuroinflammation when injected intravenously into healthy rats (Fricke et al., 2020). Balusu et al. (2016) found that peripheral inflammation also increased the number of EVs released from choroid-plexus epithelium cells into the cerebrospinal fluid which were able to pass ependymal cells and be taken up by astrocytes and microglia causing an inflammatory response in the brain. They also found that peripheral inflammation-induced changes in the proteome and miRNA of choroid-plexus epithelium EVs that were associated with the response seen in glial cells, suggesting an additional mechanism by which peripheral inflammation or infection could induce inflammation in the CNS.

Studies on the ability of EVs from virus-infected cells to cross the BBB are limited, however, Zhou et al. (2018) found that the Langat virus was secreted in arthropod EVs and able to infect human brain microvascular endothelial cells. Once infected, brain microvascular endothelial cells released EVs containing Langat virus RNA that were taken up by neurons leading to neuronal infection with the virus. Additionally, EVs from infected neurons were capable of transmitting infection to naïve neurons. This suggests EVs may be critical to viral transmission across the BBB and within the brain. Similarly, András et al. (2017) demonstrated that endothelial cells at the BBB released an increased number of EVs following exposure to HIV-1 and that those EVs were taken up by astrocytes once in the brain. In a follow-up study, they also found that exposure to HIV-1 altered the protein contents of EVs released from endothelial cells at the BBB, suggesting an additional mechanism by which the EVs may exert an effect on astrocytes or other cells of the CNS (András et al., 2020). While there is a need for further research, these studies demonstrate the potential of EVs to aid in viral infiltration of the CNS and subsequent alteration of glial phenotypes.

## Ability of EVs to Manipulate Glial Phenotypes

**Figure 2:**
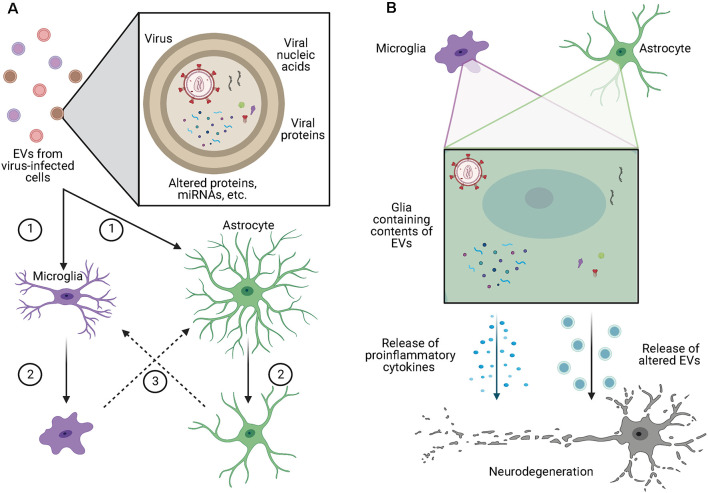
EVs alter glial phenotypes and contribute to neurodegeneration. **(A)** EVs from virus-infected cells may contain competent virus, viral nucleic acids, viral proteins, or contents which have been altered by viral infection of the cell. These EVs are (1) taken up by microglia or astrocytes causing (2) those cells to take on a more proinflammatory phenotypes which can (3) cause the activation of other surrounding glia. **(B)** Microglia and astrocytes containing the contents of EVs from virus-infected cells release proinflammatory cytokines as well as increased volumes of EVs with altered contents, both of which have been shown to cause toxic effects in neurons. Figure created with BioRender.com.

Within the brain, EVs have been shown to alter glial phenotypes through a variety of mechanisms (Figure 2A). In the context of glioblastoma, EVs enhance podosome formation and degradation of the extracellular matrix (ECM) by normal astrocytes, allowing for the expansion of the tumor while also inducing a senescence-associated secretory phenotype in those astrocytes (Hallal et al., 2019). Additionally, EVs from glioblastoma cells contain miRNA-21, are readily taken up by microglia, and cause decreased expression of miRNA-21 target genes, including Btg2, and increased proliferation (Abels et al., 2019). Along similar lines, Maas et al. (2020) found that microglia containing EVs from glioblastoma cells had reduced expression of genes involved in the ability of microglia to sense changes in the environment, potentially making them less capable of detecting and eliminating tumor cells. Additionally, the same microglia had increased expression of phagocytic receptors and ECM degrading enzymes, which may increase the clearing of debris and ECM to allow for further tumor growth. Importantly, Maas et al. (2020) found this pattern of altered gene expression was not only seen in mice, but also in microglia isolated from the core of human glioblastomas, suggesting a conserved mechanism across species.

The alteration of glial phenotypes by EVs has also been demonstrated in neuronal injury models. In a mouse model of traumatic brain injury, astrocytic EVs containing miR-873a-5p were shown to inhibit the phosphorylation of proinflammatory proteins within microglia, including NF-kB p65 and ERK, shifting the exposed microglia to a more anti-inflammatory phenotype (Long et al., 2020). Additionally, following spinal cord injury, Wang et al. (2018) found that peripheral injection of mesenchymal stem cells or EVs derived from mesenchymal stem cells were both capable of reducing the proportion of A1 astrocytes through inhibition of NF-kB activation, while also preventing the loss of synapses, myelin, and neurons. This suggests a potential anti-inflammatory therapeutic application of EVs in various neuroinflammatory conditions.

Within a viral context, it has been shown that EVs can transmit the viral infection to glial cells *via* a receptor-independent mechanism (O’Hara et al., 2020). Infection with free-virus alters glial phenotypes, suggesting that EVs from virus-infected cells may have similar effects. In support of this, Yang et al. (2018) found that microglial phenotypes were altered by interaction with EVs released from astrocytes that had been exposed to HIV. More specifically, after exposure to the HIV protein Tat, astrocyte-derived EVs containing increased miR-9 levels were taken up by microglia causing decreased expression of PTEN (involved in proliferation, migration, and cell-cycle regulation) within the microglia and increased microglial migration. This suggests a specific mechanism by which viral exposure of astrocytes can alter the behavior of microglia in an EV-dependent fashion. Similarly, dengue virus infected cells release miR-148a in EVs which induce expression of proinflammatory cytokines in microglia upon internalization (Mishra et al., 2020). While these studies provide evidence that viruses may alter glial phenotypes *via* EVs, additional research is needed to gain a broader understanding of the mechanisms involved and the potential impacts on neurodegeneration.

## EVs as A Source of Neuroinflammation and Neurodegeneration

Neuroinflammation and neurotoxicity have been associated with EVs in a variety of settings. EVs isolated from the serum of rats exposed to lipopolysaccharide or a partial hepatectomy were capable of inducing the release of proinflammatory cytokines in cerebrospinal fluid, both when administered intracerebroventricularly or peripherally to naïve rats (Fricke et al., 2020). This suggests EVs in the blood not only cross the BBB but also induce neuroinflammation once within the brain. Similarly, in a traumatic brain injury model, microglia released elevated levels of EVs that were capable of inducing neuroinflammation when administered to naïve animals (Kumar et al., 2017). When activated, microglia have also been shown to release increasing numbers of EVs (Bianco et al., 2005) which are capable of activating naïve glial cells (Verderio et al., 2012). Further, Beneventano et al. (2017) found that EVs from activated microglia had varying effects on neurotoxicity, depending on the source of microglial activation. In a study on the effects of alcoholism on the brain, ethanol was shown to increase the release of the miRNA let-7b and the TLR4 agonist high mobility group box-1 in microglia-derived EVs, both of which were associated with increased neurotoxicity (Coleman et al., 2017). Similar effects of EVs on neurodegeneration have also been demonstrated in the context of viral infections.

As neurons do not express the CD4 receptor for HIV-1, they are resistant to direct infection by the virus (González-Scarano and Martín-García, 2005), yet neuronal injury is a hallmark of HIV-associated dementia. Many mechanisms have been proposed for how this neuronal injury occurs, including *via* neuroinflammatory effects, *via* interaction with HIV-1 proteins released from other CNS cell types, or *via* uptake of EVs released from infected CNS cells. In support of the latter, Hu et al. (2012) found that exposure of astrocytes to the combination of HIV-1 protein Tat and morphine led to elevated levels of miR-29b in astrocyte-derived EVs which when taken up by neurons led to decreased viability. Additionally, when astrocytes were exposed to Tat alone, there was upregulated expression and release of miR-7 in EVs (Hu et al., 2020). The uptake of these EVs by neurons inhibited the expression of neuroligin 2 and increased synaptic loss. The HIV-1 protein Nef is also released in astrocyte-derived EVs and when taken up by neurons is correlated with several signs of neurotoxicity (Sami Saribas et al., 2018). Additionally, miR-21 is released in EVs from microglia and macrophages in the brains of SIV-infected non-human primates, and when these EVs were taken up by neurons they triggered necroptosis in a TLR7-dependent fashion (Yelamanchili et al., 2015). Another group found that glutaminase is released in macrophage and microglial EVs at elevated rates after HIV infection or lipopolysaccharide exposure (Wu et al., 2015). Importantly, the increased release of glutaminase in EVs was associated with neurotoxic effects, suggesting an additional EV-associated mechanism by which HIV infection may lead to neurotoxicity. Beyond HIV, viral effects on neurons *via* EVs have also been suggested in Zika virus (ZIKV) infections (Zhou et al., 2019), where infection of neurons with ZIKV *in vitro* was shown to increase the release of EVs that contained both viral RNA and proteins. These EVs were then capable of transmitting the active infection to naïve neurons. As active infection of neurons with ZIKV induces axonal degeneration and cell death, these findings suggest that EVs may contribute to the spread of neurodegeneration in ZIKV infections. Further, after infection with the Japanese encephalitis virus, expression of let-7a/b is increased in microglia, inducing an inflammatory phenotype and altering the content of EVs released from these cells to include let-7a/b (Mukherjee et al., 2019). When taken up by neurons, EVs containing let-7a/b subsequently cause neuronal damage through the activation of various caspases, indicating a specific mechanism by which viral infection may induce neurotoxicity *via* glial EVs (Figure 2B.

## Discussion

EVs are emerging as important players in various disorders of the CNS including glioblastoma, neuronal injury, and viral infection. Viruses and viral proteins have been found within EVs, allowing for evasion of the immune response and increased cellular tropism. EVs can also cross the BBB in a variety of ways (Figure 1), suggesting a mechanism for viruses to infiltrate the CNS. Within the brain, EVs from virus-infected cells can alter glial phenotypes and induce neurotoxic effects through the transfer of viruses, viral proteins, or altered miRNA or protein content (Figure 2). While much of the research related to viral manipulation of EVs within the CNS is limited to HIV, this research provides proof of concept and suggests a mechanism by which other viruses, including SARS-CoV-2, may alter glial and neuronal function leading to cognitive deficits. This is a promising area of research that is in need of additional exploration.

Another emerging area of research related to EVs is the potential for brain-derived EVs to serve as biomarkers of disease (Thompson et al., 2016). For instance, a correlation between the concentration of microglia/macrophage-derived EVs in the cerebrospinal fluid and severity of disease was shown in a mouse model of multiple sclerosis (Verderio et al., 2012). Similarly, an association was found between HIV infection and the abundance of brain-derived EVs in the serum of rats (Dagur et al., 2020) and in the cerebrospinal fluid of humans (Guha et al., 2019). As methods for isolating specific subsets of EVs continue to improve (McNamara and Dittmer, 2020), so too should the identification of specific biomarkers indicative of various neuronal and glial alterations (Pulliam et al., 2019). This could serve as a valuable, non-invasive tool for the diagnosis and monitoring of many neurological disorders that are otherwise difficult to track.

Overall, while EVs are valuable tools for intercellular communication in a healthy individual, their ability to be manipulated by viruses raises many questions about their role in viral infiltration of the CNS and subsequent alteration of resident brain cells. Current data support a pro-viral role of EVs in these processes leading to neuroinflammation and neurodegeneration, but much is yet to be uncovered.

## Author Contributions

MH and AM conceptualized the manuscript and figures. MH conducted the literature search and designed and drafted the manuscript and figures. AM critically revised and edited the manuscript and figures. All authors contributed to the article and approved the submitted version.

## Conflict of Interest

The authors declare that the research was conducted in the absence of any commercial or financial relationships that could be construed as a potential conflict of interest.
